# The role of temporal predictability for early attentional adjustments after conflict

**DOI:** 10.1371/journal.pone.0175694

**Published:** 2017-04-14

**Authors:** Klaas Bombeke, Zachary D. Langford, Wim Notebaert, C. Nico Boehler

**Affiliations:** Department of Experimental Psychology, Ghent University, Ghent, Belgium; University of Akron, UNITED STATES

## Abstract

A frequently-studied phenomenon in cognitive-control research is conflict adaptation, or the finding that congruency effects are smaller after incongruent trials. Prominent cognitive control accounts suggest that this adaptation effect can be explained by transient conflict-induced modulations of selective attention, reducing congruency effects on the next trial. In the present study, we investigated these possible attentional modulations in four experiments using the Stroop and Flanker tasks, dissociating possible enhancements of task-relevant information from suppression of task-irrelevant information by varying when this information was presented. In two experiments, the irrelevant stimulus information was randomly presented shortly before, at the same time, or briefly after the presentation of the relevant dimension. In the other two, irrelevant information was always presented first, making this aspect fully predictable. Despite the central role that attentional adjustments play in theoretical accounts of conflict adaption, we only found evidence for such processes in one of the four experiments. Specifically, we found a modulation of the attention-related posterior N1 event-related potential component that was consistent with paying less attention to the irrelevant information after incongruent trials. This was accompanied by increased inter-trial mid-frontal theta power and a theta-power conflict adaptation effect. We interpret these results as evidence for an adaptive mechanism based on relative attentional inhibition. Importantly, this mechanism only clearly seems to be implemented in a very specific context of high temporal predictability, and only in the Flanker task.

## Introduction

Cognitive control involves the ability to detect conflicting cues in the environment and to adjust our information processing system in order to optimize behavioral responses. These control adjustments invoked by conflict have sparked a lot of scientific interest [for a review, see 1, 2]. The “Gratton effect”, or conflict adaptation effect, is the hallmark of such research, describing the phenomenon that conflict effects are attenuated after incongruent trials [3]. Despite alternative accounts [4, 5], the traditionally most accepted explanation for this effect comes from the model of Botvinick and colleagues [6], explaining it through a monitoring operation wherein the detection of conflict triggers a transient increase in selective attention, reducing the amount of conflict experienced in the next trial [6–9]. Although there has been an abundance of studies investigating psychological or pathology-related modulations of conflict adaptation effects [10, 11], some procedural aspects remain unclear, especially regarding the role of sensory modulations as they unfold rapidly in time. Previous research on this question has predominantly used fMRI, revealing modulations in prefrontal control structures [7, 12], which trigger subsequent reductions in motor readiness [13–15] and/or modulations of sensory processing [13, 14, 16]. When investigating the role of sensory adjustments, anatomical distinctions between specialized sensory processing modules [e.g. faces, 13, 14–17] are usually used. Some of these studies have related this adaptation effect to enhanced processing of the relevant stimulus dimension on n+1 trials [16], whereas others also found inhibition of irrelevant stimuli [17].

However, fMRI studies are limited by their temporal resolution and EEG studies have not addressed this question to the same extent. Among the few studies to date, Scerif et al. [18] showed a selective enhancement of the visual P1 component for incongruent trials when preceded by incongruent trials in a flanker task with simultaneously presented distractor and target arrows. Interestingly, for no-target flanker trials following incongruent trials, they observed a smaller P1 component. Assuming that conflict detection leads to increased suppression of flanker arrows, this smaller P1 component could be explained as more focused spatial attention. Later, Suzuki and Shinoda [19] observed decreased N1 amplitudes for Flanker trials preceded by incongruent stimuli compared to trials preceded by congruent stimuli. This decrease in attentional allocation was correlated with a larger increase in frontal alpha activity, most likely related to proactive frontal control mechanisms. Taken together, these two studies seem to be in line with attentional adjustments after conflict. Yet, these are the only two human EEG studies we are aware of that explicitly look at attentional mechanisms in a sequential conflict-adaptation context, which is surprising given their theoretical prominence. Moreover, their results differ on the level of which ERP component is affected, and only one of them tried to tap into the specificity of enhancement of relevant vs. suppression of irrelevant information.

A possible pitfall when studying post-conflict adjustments is the possibility of simultaneous enhancement of relevant and suppression of irrelevant information, which might camouflage each other in the scalp EEG using standard paradigms not optimized for such distinctions. Here, we further investigate the nature of attentional modulations during conflict adaptation in a serial reaction time context with a paradigm that has generally been shown to index attentional allocation separately to the relevant and irrelevant stimulus dimension of a conflicting stimulus. The present paradigm was derived from a study employing Stroop stimuli wherein the word and the color component were presented with different stimulus-onset asynchronies (SOAs), varying between -200 ms (word first) to +200 ms (color first) [20]. This study compared blocks where this temporal arrangement was constant versus random, which revealed dissociations for behavioral and EEG markers of conflict. More importantly for the present work, the comparison of the EEG data for the -200 ms condition between these blocks also yielded evidence for an attentional modulation that preceded the presentation of the relevant stimulus dimension, yielding a smaller negativity in the constant-SOA blocks starting approximately 150 ms after the onset of this stimulus dimension. This modulation, due to timing and spatial distribution, was related to a selection negativity [SN; 21] and was thus interpreted as indexing the degree to which attention was deployed to the word component. Importantly, these data suggest that with temporal predictability, participants are better at activating temporary selective filters in line with temporal orienting ideas [e.g. 22].

In the present study, we hypothesized that a similar posterior modulation could also be present as a neural marker of attentional adjustments in the conflict adaptation effect in conditions with temporal predictability (i.e., when the irrelevant information was systematically presented before the relevant information). This prediction fits with recent proactive accounts of conflict adaptation. Duthoo et al. [23] for instance demonstrated that participants’ predictions about the upcoming (in)congruency influence conflict adaptation. However, Jiménez and Méndez [24, 25] came to a different conclusion, finding that conflict adaptation mostly depends on the average of experienced conflict in previous trials and not on the participants' expectancies.

Taken together our goals of studying neural attentional markers of conflict adaptation that can be both driven by reactive and proactive control processes, we conducted four experiments with different groups of participants. Based on Appelbaum et al. [20], we started with two experiments using the Stroop task, but ultimately extended our approach also to the Flanker task, given the study of Scerif et al. [18] and Suzuki and Shinoda [19] with a P1 and N1 modulation in the Flanker task, respectively. For each type of conflict task, we had one experiment in which the irrelevant stimulus dimension (word color in the Stroop task or distractor arrows in the Flanker task) was randomly presented before, after, or simultaneous with the relevant stimulus (ink color or target arrow), and one experiment in which the irrelevant information was always presented before the relevant stimulus. This set of experiments developed sequentially based on the fact that we had anticipated finding evidence for attentional adjustments related to conflict adaptation in our first experiment, but failed to do so for most experiments, hence prompting us to run a set of four experiments combining the factors of task (Stroop vs. Flanker) and temporal predictability. Furthermore, we planned to explore the context of possible attentional modulations by looking at oscillatory activity before and after the second trial in a conflict adaption sequence, in order to relate to earlier work showing oscillatory power modulations between the two trials [15, 26–28] and conflict-related frontal modulations in consecutive trials [29–32].

## Methods

Given the similarities between the four experiments with respect to design, acquisition and analysis, the sections below describe common aspects while explicitly pointing out differences.

### Participants

For each experiment, participants (experiment 1: *n* = 23, 9 ♂, 14 ♀; experiment 2: *n* = 23, 10 ♂, 13 ♀; experiment 3: *n* = 22, 8 ♂, 14 ♀; experiment 4: *n* = 22, 10 ♂, 12 ♀, ranging between 18–26 years) were selected on the basis of an online prescreening questionnaire via the Experimetrix scheduling system (https://experimetrix2.com/rug/). In these questionnaires, people only had to indicate their age, gender, handedness and if they had abnormal vision or any neurological disorders. Every interested candidate below 30 years old without abnormal vision (corrected vision was allowed) and neurological disorders would be invited to subscribe for the experiment via the scheduling system. Before completing the experiment, participants signed an informed consent in which they were informed about their right to stop the experiment whenever they wanted. The procedures were approved by the ethical committee of the Faculty of Psychology and Educational Sciences of Ghent University and participants received 25€ for a session that lasted two hours.

### Stimuli

In experiment 1 and 2, the paradigm was based on Appelbaum et al. [20]. In their variant of the Stroop task, red-, green-, blue- and yellow-colored rectangular boxes overlaid with the color-words "RED","GREEN","BLUE" or "YELLOW" were presented on a gray background with a small fixation dot at the center of the screen. The first independent variable was congruency, so trials could be either congruent (e.g., RED on a red box) or incongruent (e.g., RED on a blue box). In each block, half of the trials were congruent (four different pairings) and half of the trials were incongruent (twelve possible pairings, distributed evenly). The second independent variable was the SOA between the relevant and irrelevant information. In experiment 1, irrelevant information was presented either 200 ms before, at the same or 200 ms after the presentation of the relevant information ("unpredictable timing"). In experiment 2, irrelevant word information was always presented 200 ms before the relevant color information ("predictable timing"). Experiments 3 and 4 used a variant of the Flanker task with arrows pointing in four different directions (left up, left down, right up, right down). White arrows were presented on a gray background with a small fixation dot at the center of the screen. Other than that, everything was exactly the same as in experiment 1 and 2, respectively, with experiment 3 using the temporal arrangement of experiment 1 and experiment 4 that of experiment 2.

### Procedure and design

**Fig 1:**
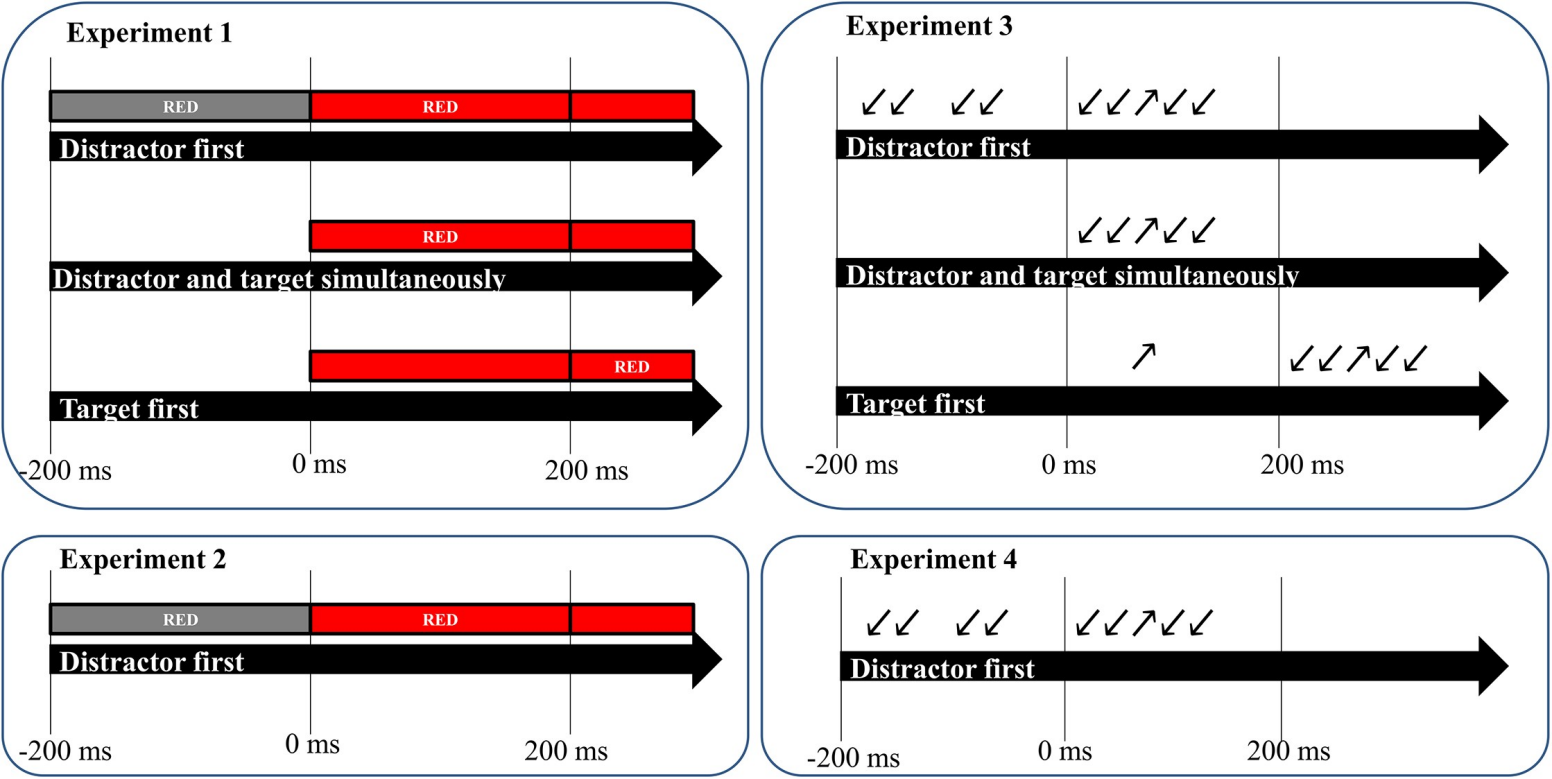
Design of the four experiments. In the Stroop experiments (experiments 1 and 2), participants were instructed to manually indicate the ink color of the rectangular box as fast and accurate as possible, while in the Flanker experiments (experiments 3 and 4) they were instructed to respond to the direction of the target arrow. In the experiments with unpredictable design, irrelevant information could be presented 200 ms before, 200 ms after or simultaneous with the relevant information, while the experiments with predictable design, irrelevant information was always presented first (again by 200 ms).

In experiment 1 and 2 (the Stroop experiments), participants were instructed to manually indicate the ink color of the rectangular box as fast and accurate as possible, while in experiment 3 and 4 (the Flanker experiments) they were instructed to respond to the direction of the target arrow (Fig 1). In the experiments with predictable timing, participants knew that the irrelevant word or distracting arrows would be presented first, while they could not foresee this in the experiments with unpredictable timing. They had to respond by manually pressing one of four keys on the keyboard corresponding to four possible colors or four different arrow directions and they had some time to memorize this response mapping before the start of the experimental phase. Responses were registered until 1300 ms after stimulus onset and there was a jittered inter-trial interval ranging from 900 to 1200 ms. Participants completed 16 pseudo-randomized blocks of 72 trials in experiment 1 and 3 and 10 blocks with the same number of trials in experiment 2 and 4. Every two blocks, participant could take a break. Since we were interested in sequential effects, we chose to have a completely randomized sequence of trials. As a consequence, the proportion of congruent trials preceded by congruent trials (CC), incongruent trials preceded by congruent trials (CI), congruent trials preceded by incongruent trials (IC) and incongruent trials preceded by incongruent trials (II) was always 25%, resulting in 288 trials per cell in experiment 1 and 3 (irrelevant-first, simultaneous and relevant first trials) and 180 trials per cell in experiment 2 and 4 (only irrelevant-first trials).

### Behavioral data acquisition and analysis

For RT analyses, the first trial of each block and incorrect or missed responses on trial n and n-1 were excluded and an outlier rejection criterion of 2 SDs was applied. RT and error rates were analyzed with repeated-measures ANOVAs (rANOVAs), with factors SOA (3 levels), previous congruency (2 levels) and current congruency (2 levels) in experiment 1 and 3, and factors previous and current congruency (each 2 levels) in experiment 2 and 4. In case of significant interactions in experiment 1 and 3, additional rANOVAs and paired samples t-tests were performed on the conflict adaptation effect (i.e. the interaction between previous and current congruency) per SOA condition. The significance threshold was set to a p-value of .05 and, when applicable, adjusted using the Greenhouse–Geisser correction for non-sphericity. Additional outlier removal procedures and/or participant exclusions are described per experiment.

### EEG acquisition, preprocessing and analysis

The EEG was acquired with a Biosemi ActiveTwo measurement system (BioSemi, Amsterdam, Netherlands), using 64 Ag-AgCl scalp electrodes attached to a standard international 10–20 system cap. Six additional external electrodes were attached to the head: left and right mastoids, which were used for later offline re-referencing, a bilateral electro-oculogram (EOG) pair next to the outer canti of the eyes to measure horizontal eye-movements and two electrodes above and below the left eye to measure vertical eye movements. Signals were amplified and digitized with a sampling rate of 512 Hz. Next, EEG data was processed using EEGLAB [33] and the ERPLAB plugin [34], both MATLAB-based. We used a bandpass filter of 0.01–30 Hz on the continuous EEG data. Epochs were always time-locked to the onset of the information that came first, depending on the condition (irrelevant distractors in irrelevant-first condition, both the irrelevant distractors and relevant target in the simultaneous condition and the relevant target in the relevant-first condition). Just like in the behavioral analyses, only trials with accurate performance on trial N-1 and N were included. Epochs included a 200 ms pre-stimulus period that was used for baseline correction and lasted 1000 ms. Trials with drifts larger than -/+ 200 μV were rejected, leading to a rejection of 5% of the epochs on average. Next, epochs were averaged within and subsequently across participants.

To test for evidence for differential attentional processing of the stimuli between post-congruent and post-incongruent trials and the interaction with different SOA conditions in experiment 1 and 3, we probed for task differences in the -200 ms window in the relevant-first, simultaneous and irrelevant-first condition (time-locked to the onset of the relevant target). The first and third condition provided an uninterrupted window for 200 ms, during which no other overlapping stimulus response activity would be present. Based on previous research on attentional selection [21] and the study of Appelbaum et al. [20], we probed the response amplitudes over ROIs comprised of left posterior sensors PO3, P3 and P1, and right posterior sensors PO4, P4 and P2. Since all stimuli were presented centrally, we did not expect lateralized effects and hence decided to collapse across the left and right posterior ROI for plotting and analyses purposes. Time windows for measurement were based on Appelbaum et al. [20] and visual inspection of the averaged ERP.

In order to probe for theta power modulations, we also performed event-related spectral perturbation analyses. Epoched data (from -500 to 2500 ms) was transformed to the frequency domain using EEGLAB's "newtimef" function [33] and baseline correction was performed using the 500 ms window before the onset of trial n-1 for inter-trial analyses and the 500 ms window before trial n for the current trial analyses. Fifty frequencies between 1 and 30 Hz were sampled uniformly between -250 and 2250 ms, with 200 sample points in between. We considered 4–8 Hz as the theta range and used the average across the different frequencies for all statistical analyses. In order to measure preparatory activity in the inter-trial interval, we took the interval between 500 and 1000 ms after the response as measurement window. By doing so, we measured oscillatory activity in the 500 ms window before the onset of the next trial (taking temporal jitter into account). Based on visual inspection of the average response across conditions and its temporal proximity to the onset of the response, we chose the 500–600 ms window after stimulus onset in trial n for the measurement of the theta power conflict adaptation effect.

Amplitudes, latencies and power measurements were statistically compared using repeated-measures ANOVAs in experiment 1 and 3 and paired samples t-tests in experiment 2 and 4. For selected analyses, we also reported Bayes factor in order to indicate how likely the absence of an effect was. Current congruency was not included in the analysis, because all measured activity in the window from 0 to 200 ms could only be related to previous congruency (the congruency status of the current trial was not yet determined, since the other dimension only appeared after 200 ms). At latencies beyond ∼250 ms, the processing of the second stimulus would begin and would overlap and distort the ERP activity evoked in response to the first one.

## Results

### Experiment 1—Stroop task with unpredictable timing

#### Behavior: Reaction time

**Fig 2:**
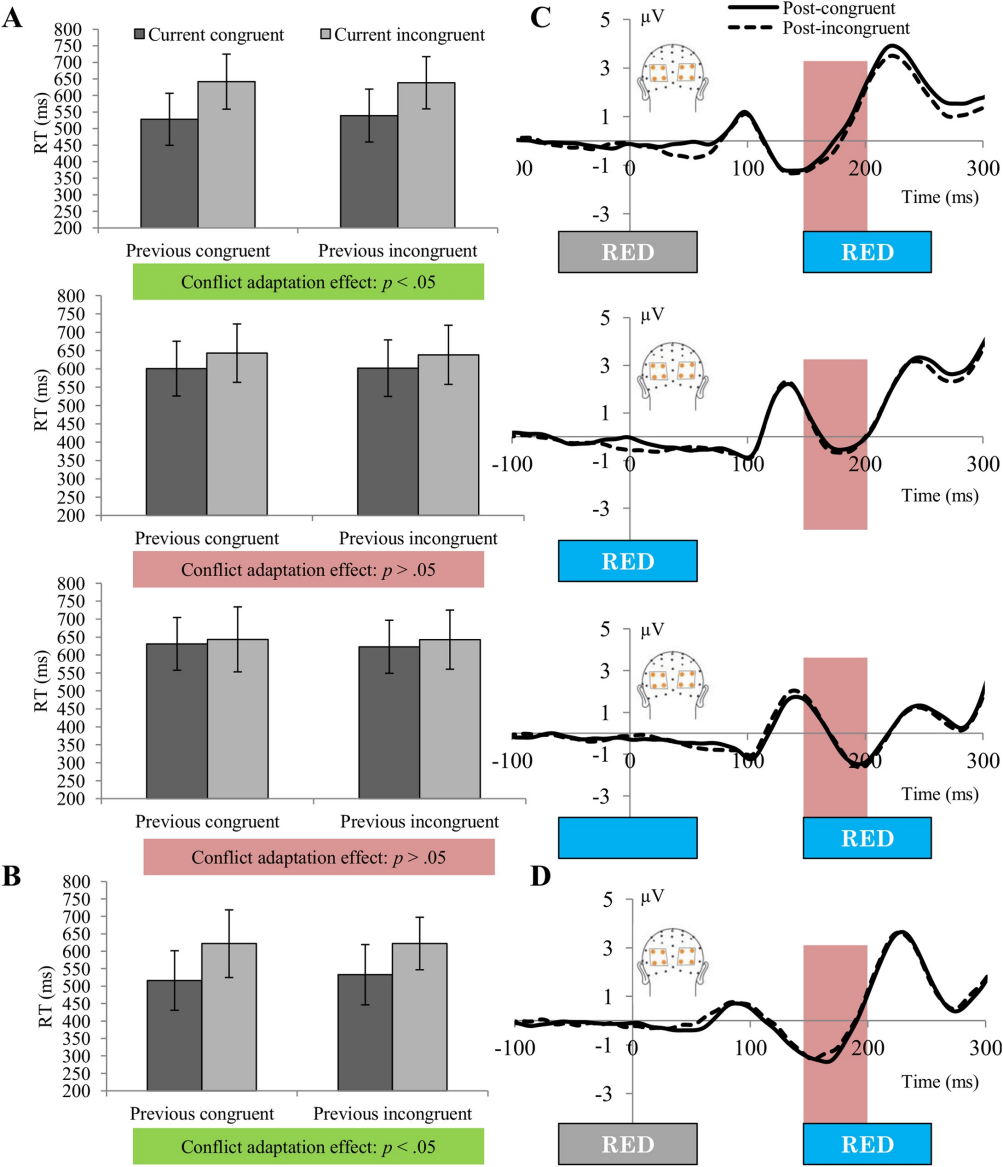
Results of experiment 1&2—Stroop experiments with predictable and unpredictable timing. (A)(B) Mean reaction times per SOA condition (irrelevant-first, simultaneous and relevant-first in experiment 1, (A); irrelevant-first in experiment 2, (B)) for current congruent (dark grey) and incongruent (light grey) trials as a function of the congruency level of the previous trial in the Stroop experiments (error bars represent 1 standard deviation around the mean). (C)(D) Early attentional ERP amplitudes (μV) per SOA condition as a function of the congruency level of the previous trial in the Stroop experiments with unpredictable (C) and predictable (D) timing. Measurements were performed for averaged activity over a 50-ms window between 150 and 200 ms post-stimulus onset for the collapsed posterior ROIs, indicated in red (left posterior sensors PO3, P3 and P1, and right posterior sensors PO4, P4 and P2). The irrelevant-first and relevant-first conditions provided an uninterrupted window for 200 ms, during which no other overlapping stimulus response activity would be present. The results show no significant ERP modulations by previous congruency.

The main effects of SOA and current congruency were significant, *F*(2,44) = 64.3, *p* < .001, *r* = .86 and *F*(1, 22) = 221.48, *p* < .001, *r* = .95, respectively, whereas the main effect of previous congruency was not, *F*(1, 22) = .1, *p* >.5, *r* = .06. As expected, trials in which irrelevant distracter information preceded the relevant target resulted in the fastest response, followed by simultaneous and relevant-first trials and participants responded in general faster to congruent trials than to incongruent trials. There was a significant interaction between SOA and current congruency, *F*(2,44) = 69.65, *p* < .001, *r* = .87, showing that the congruency effect on trial n was largest for trials in which the distracter preceded the target and smallest for trials in which the target preceded the distracter. The interaction between SOA and previous congruency did not reach significance, *F*(2, 44) = 1.27, *p* >.2, *r* = .22. An overall conflict adaptation effect, as reflected in the interaction between previous congruency and current congruency, was not present, *F*(1,22) = .68, *p* >.5, *r* = .17. Importantly, the three-way interaction between SOA, previous congruency and current congruency was only marginally significant, *F*(2, 44) = 2.56, *p* = .09, *r* = .32. Because we were mostly interested in the conflict adaptation effect split out per SOA condition, we looked at the interaction between previous and current congruency per condition. The conflict adaptation effect was significant for trials in which the irrelevant word was presented first, *F*(1,22) = 5.40, *p* < .05, *r* = .44, so the congruency effect was smaller after incongruent trials (99.15 ms) than after congruent trials (113.75 ms). Somewhat surprisingly, for trials where the relevant and irrelevant dimension were presented at the same time, the conflict adaptation effect was not significant, *F*(1,22) = .51, *p* > .4, *r* = .15. Also for the condition in which relevant information was presented first, we could not find a significant conflict adaptation effect, *F*(1,22) = .73, *p* >.4, *r* = .18 (Fig 2A & Table 1).

**Table 1:** Descriptive statistics of reaction times (percentage correct) of the behavioral data of the Stroop experiment with unpredictable design (CC: congruent followed by congruent, CI: congruent followed by incongruent, IC: incongruent followed by congruent, II: incongruent followed by incongruent).

	Irrelevant first		Simultaneous		Relevant first	
Method	Mean	SD	Mean	SD	Mean	SD
CC	528 ms (93%)	79 ms (5%)	601 ms (93%)	75 ms (5%)	631 ms (92%)	74 ms (5%)
CI	642 ms (89%)	83 ms (7%)	643 ms (91%)	80 ms (7%)	644 ms (94%)	90 ms (4%)
IC	539 ms (94%)	80 ms (4%)	602 ms (93%)	77 ms (6%)	623 ms (94%)	74 ms (4%)
II	638 ms (89%)	79 ms (7%)	638 ms (92%)	81 ms (6%)	643 ms (93%)	82 ms (5%)

#### Behavior: Error rate

On average, participants made an error in 9.23% of the trials. The main effects of SOA and current condition were significant, *F*(2,44) = 6.26, *p* = .004, *r* = .47 and *F*(1, 22) = 14.78, *p* = .001, *r* = .63, respectively, whereas the main effect of previous congruency was not, *F*(1, 22) = 1.17, *p* = .29, *r* = .23. Participants made the most errors in trials where the irrelevant information was presented first (8.65%), compared to trials with simultaneous (7.59%) and relevant first (6.59%) presentation. There was a significant interaction between SOA and current congruency, *F*(2,44) = 5.18, *p* = .01, *r* = .44, showing that the congruency effect on trial n was largest for trials in which the distracter preceded the target and smallest for trials in which the target preceded the distracter, but no interaction between SOA and previous congruency, *F*(2, 44) = .03, *p* = .97, *r* = .03. An overall conflict adaptation effect, as reflected in the interaction between previous congruency and current congruency, was not present, *F*(1,22) = .71, *p* = .41, *r* = .18. Also the three-way interaction between SOA, previous congruency and current congruency did not reach significance, *F*(2, 44) = 2.04, *p* = .14, *r* = .29.

#### ERPs

A repeated-measures ANOVA with factors previous congruency and current SOA performed over a 50-ms window between 150 and 200 ms post-stimulus onset for the collapsed posterior ROIs revealed no significant main effects for current SOA and previous congruency, *F*(2,44) = .26, *p* > .1, *r* = .11 and *F*(1,22) = .58, *p* >.1, *r* = .16, respectively. Also the interaction between SOA and previous congruency did not reach significance, *F*(2,44) = 1.26, *p* > .1, *r* = .23. Therefore, we did not look at the isolated effect of previous congruency per SOA condition. However, we did calculate inverse Bayes factors (BF_01),_ allowing us to report the likelihood of the absence of the effect. The inverse Bayes factors for the effect of previous congruency on irrelevant-first (BF_01_ = 11.82), simultaneous (BF_01_ = 3.78) and relevant-first trials (BF_01_ = 4.47) showed 'strong', 'substantial' and 'substantial' evidence for the null hypothesis, respectively [35]. As can be seen in Fig 2C, there was a difference in posterior activity between 25 ms and 75 ms between post-congruent and post-incongruent trials for irrelevant-first trials. Since it is highly unlikely that activation differences that early can be related to the time-locked event, we interpret this difference as an artifact in the grand average. Overall, it seems clear that there was no early posterior modulation in trial n related to the congruency of trial n-1.

### Experiment 2—Stroop task with predictable timing

#### Behavior: Reaction time

There was no main effect of previous congruency, *F*(1,22) = 1.77, *p* = .20, *r* = .27, but a highly significant main effect of current congruency, *F*(1,22) = 236.33, *p* < .001, *r* = .96 (II trials = 622.17 ms; IC trials = 532.72 ms; CC trials = 516.06 ms; CI trials = 621.72 ms). Also the interaction between previous congruency and current congruency, *F*(1,22) = 5.09, *p* = .03, *r* = .42, was significant, indicating the presence of a behavioral conflict adaptation effect (Fig 2B & Table 2). The congruency effect was smaller after incongruent trials (89 ms) than after congruent trials (105 ms).

**Table 2:** Descriptive statistics of reaction times (percentage correct) of the behavioral data of the Stroop experiment with predictable design.

	Irrelevant first	
Method	Mean	SD
CC	516 ms (91%)	85 ms (5%)
CI	622 ms (84%)	97 ms (19%)
IC	533 ms (93%)	86 ms (4%)
II	622 ms (86%)	75 ms (18%)

#### Behavior: Error rate

On average, participants made an error in 11.24% of the trials. We both found a main effect of previous congruency and current congruency, *F*(1,22) = 9.07, *p* = .01, *r* = .54, and *F*(1,22) = 4.46, *p* = .05, *r* = .41, respectively (II trials = 13.82%; IC trials = 6.91%; CC trials = 8.52%; CI trials = 15.70%). The interaction between previous congruency and current congruency was not significant, *F*(1,22) = .07, *p* = .80, *r* = .05, indicating the absence of an conflict adaptation effect for the error rates.

#### ERPs

T-tests performed over the averaged 50 ms window between 150 and 200 ms post-stimulus onset for the collapsed posterior ROIs revealed no significant differences between post-congruent (M = -.90, SD = 3.03) and post-incongruent trials (M = -.73, SD = 2.89), *t*(22) = -0.88, *p* = .39. Just like in the unpredictable design, we could not observe a posterior attentional modulation as neural marker of conflict adaptation for any of the conditions (Fig 2D). An inverse Bayes factor of BF_01_ = 2 supported 'anecdotal' evidence in favor of the null hypothesis.

### Experiment 3—Flanker task with unpredictable timing

#### Behavior: Reaction time

**Fig 3:**
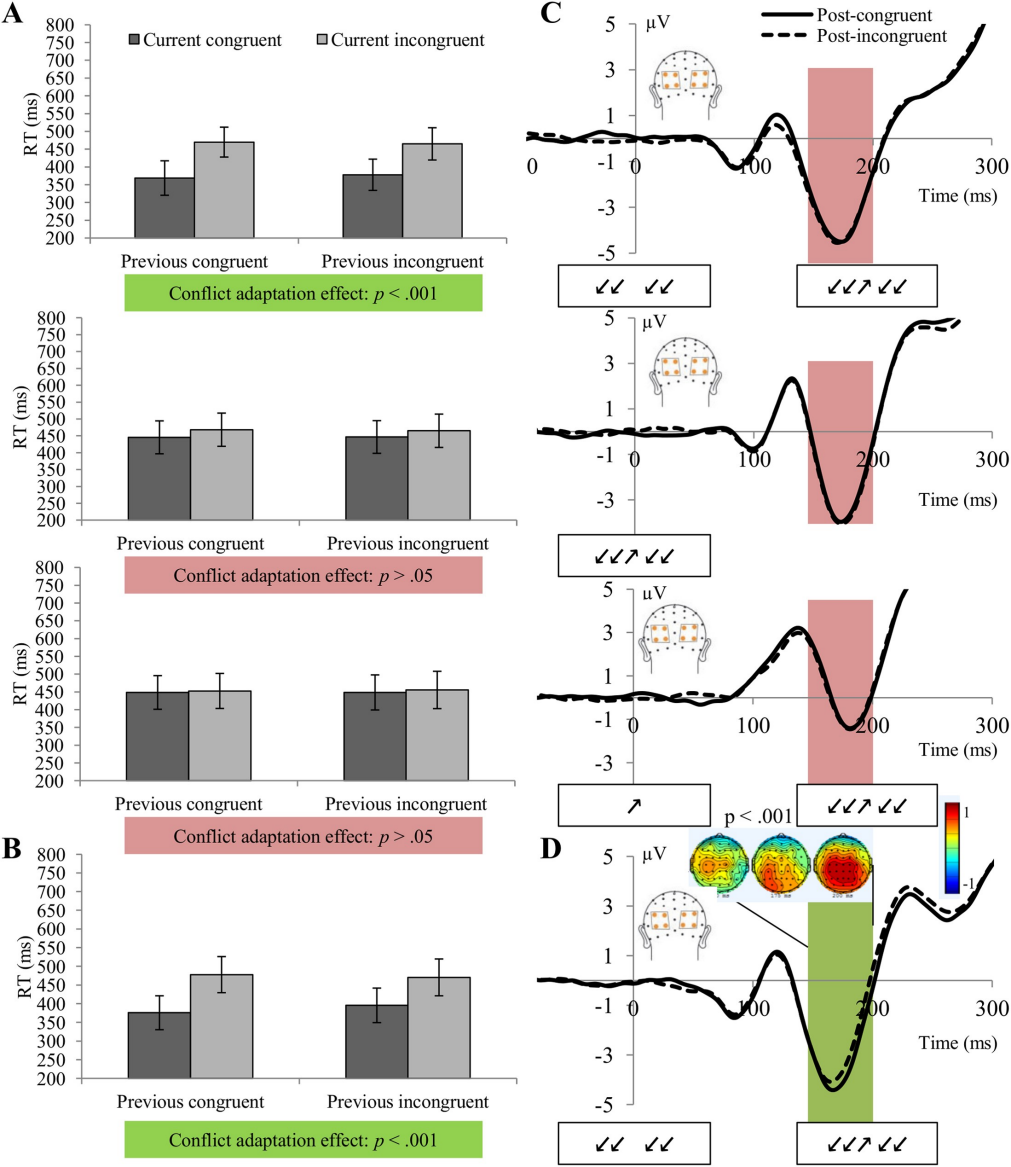
Results of experiment 3&4—Flanker experiments with predictable and unpredictable timing. (A)(B) Mean reaction times per SOA condition (irrelevant-first, simultaneous and relevant-first in experiment 1, (A); irrelevant-first in experiment 2, (B)) for current congruent (dark grey) and incongruent (light grey) trials as a function of the congruency level of the previous trial in the Flanker experiments (error bars represent 1 standard deviation around the mean). (C)(D) Early attentional ERP component amplitudes (μV) per SOA condition as a function of the congruency level of the previous trial in the Flanker experiments with unpredictable (C) and predictable (D) timing. Measurements were performed for averaged activity over a 50-ms window between 150 and 200 ms post-stimulus onset for the collapsed posterior ROIs, indicated in red (left posterior sensors PO3, P3 and P1, and right posterior sensors PO4, P4 and P2). The irrelevant-first and relevant-first conditions provided an uninterrupted window for 200 ms, during which no other overlapping stimulus response activity would be present. Only the Flanker task with a predictable irrelevant-first temporal arrangement showed a significant posterior modulation reflecting a decreased negativity starting around 150 ms, likely representing decreased early attentional processing.

The main effects of SOA and current congruency were significant, *F*(1.36,29.91) = 134.51, *p* < .001, *r* = .93 and *F*(1, 21) = 554.01, *p* < .001, *r* = .98, respectively, whereas the main effect of previous congruency was not, *F*(1, 21) = .1, *p* >.3, *r* = .20. Participants responded faster to irrelevant-first trials compared to simultaneous and relevant-first trials. There was a significant interaction between SOA and current congruency, *F*(1.49,32.80) = 221.82, *p* < .001, *r* = .95, showing that the congruency effect on trial n was largest for trials in which the distracter preceded the target and smallest for trials in which the target preceded the distracter. The interaction between SOA and previous congruency did not reach significance, *F*(2, 42) = 1.65, *p* >.2, *r* = .26. An overall conflict adaptation effect, as reflected in the interaction between previous congruency and current congruency, was present, *F*(1,21) = .7.85, *p* < .05, *r* = .51. Importantly, the three-way interaction between SOA, previous congruency and current congruency was highly significant, *F*(1, 42) = 11.23, *p* < .001, *r* = .58. The conflict adaptation effect was significant for trials in which the irrelevant word was presented first, *F*(1,21) = 20.61, *p* < .001, *r* = .7: the congruency effect was smaller after incongruent trials (86 ms) than after congruent trials (101 ms). For trials where the relevant and irrelevant dimension were presented at the same time, the conflict adaptation effect was not significant, *F*(1,21) = 2.50, *p* >.1, *r* = .32. Also for the condition in which relevant information was presented first, we could not find a significant conflict adaptation effect, *F*(1,21) = 1.15, *p* >.2, *r* = .22 (Fig 3A and Table 3).

**Table 3:** Descriptive statistics of reaction times (percentage correct) of the behavioral data of the Flanker experiment with unpredictable design.

	Irrelevant first		Simultaneous		Relevant first	
Method	Mean	SD	Mean	SD	Mean	SD
CC	369 ms (99%)	48 ms (1%)	446 ms (96%)	49 ms (3%)	448 ms (96%)	47 ms (4%)
CI	470 ms (83%)	42 ms (12%)	468 ms (96%)	49 ms (4%)	453 ms (95%)	49 ms (4%)
IC	378 ms (98%)	44 ms (2%)	447 ms (96%)	48 ms (4%)	448 ms (97%)	49 ms (3%)
II	465 ms (87%)	45 ms (11%)	465 ms (96%)	49 ms (4%)	455 ms (97%)	52 ms (3%)

#### Behavior: Error rate

On average, participants made an error in 5.18% of the trials. The main effects of SOA, current condition and previous condition were significant, *F*(2,42) = 25, *p* < .001, *r* = .73, *F*(1, 21) = 40.89, *p* < .001, *r* = .81 and *F*(1,21) = 9.89, *p* = .005, *r* = .56, respectively. Participants again made the most errors in trials where the irrelevant information was presented first (8.06%), compared to trials with simultaneous (3.84%) and relevant first (3.63%) presentation. There was a significant interaction between SOA and current congruency, *F*(1.16,25.4) = 30.43, *p* < .001, *r* = .76, indicating that the congruency effect on trial n was largest for distracter-first trials, followed by target-first and simultaneous trials. The interaction between SOA and previous congruency did not reach significance, *F*(2, 42) = 2.73, *p* = .08, *r* = .33. An overall conflict adaptation effect, as reflected in the interaction between previous congruency and current congruency, was present, *F*(1,21) = 7.66, *p* = .01, *r* = .51. Also the three-way interaction between SOA, previous congruency and current congruency did reach significance, *F*(1.50, 33.02) = 4.16, *p* = .04, *r* = .40. Splitting out this conflict adaptation effect for the different SOA conditions resulted in a significant effect for trials in which the irrelevant word was presented first, *F*(1,21) = 9.24, *p* = .006, *r* = .54: the congruency effect was smaller after incongruent trials (10.74% errors) than after congruent trials (15.17% errors). For trials in which the relevant and irrelevant dimension were presented at the same time, the conflict adaptation effect was not significant, *F*(1,21) = .21, *p* = .65, *r* = .1. Also for the condition in which relevant information was presented first, we did not observe a significant conflict adaptation effect, *F*(1,21) = .28, *p* = .60, *r* = .11.

#### ERPs

A repeated-measures ANOVA with factors previous congruency and current SOA performed over the averaged 50 ms window between 150 and 200 ms post-stimulus onset for the collapsed posterior ROIs revealed a significant main effect for current SOA, but not for previous congruency, *F*(2,42) = 36.7, *p* < .001, *r* = .80 and *F*(1,21) = .43, *p* > .5, *r* = .14, respectively. The main effect of current SOA showed the largest ERP response for trials in which the distracter preceded the target, followed by simultaneous and target-first trials. The interaction between SOA and previous congruency did not reach significance, *F*(2,42) = .06, *p* > .5, *r* = .05 (Fig 3C). The inverse Bayes factors for the effect of previous congruency on irrelevant-first (BF_01_ = 11.82), simultaneous (BF_01_ = 3.78) and relevant-first trials (BF_01_ = 4.47) all indicated 'substantial' evidence for the null hypothesis in all three conditions [35].

Visual inspection of the stimulus-locked ERP seems to suggest a P1 modulation in the irrelevant-first condition. However, when statistically comparing the average amplitude between 100 and 150 ms in this condition only, a paired samples t-test showed this modulation was not significant, *t*(21) = 1.32, *p* >.2.

### Experiment 4—Flanker task with predictable timing

#### Behavior: Reaction time

There was a main effect of previous congruency, *F*(1,21) = 15.28, *p* < .001, *r* = .64 and current congruency, *F*(1,21) = 165.55, *p* < .001, *r* = .94. Also the interaction between previous congruency and current congruency was highly significant, *F*(1,21) = 56.234, *p* < .001, *r* = .85, indicating the presence of a behavioral conflict adaptation effect (Fig 3B). The congruency effect was consistently smaller after incongruent trials (75 ms) than after congruent trials (102 ms) (Fig 3B and Table 4)

**Table 4:** Descriptive statistics of reaction times (percentage correct) of the behavioral data of the Flanker experiment with predictable design.

Method	Mean	SD
CC	376 ms (95%)	45 ms (4%)
CI	478 ms (85%)	48 ms (11%)
IC	395 ms (88%)	46 ms (9%)
II	470 ms (81%)	49 ms (15%)

#### Behavior: Error rate

On average, participants made an error in 12.95% of the trials, and there were significant main effects of previous congruency and current congruency, *F*(1,21) = 15.93, *p* = .001, *r* = .65, and *F*(1,21) = 20.49, *p* < .001, *r* = .70, respectively (II trials = 18.91%; IC trials = 12.35%; CC trials = 5.22%; CI trials = 15.30%). Also the interaction between previous congruency and current congruency was significant, *F*(1,21) = 5.20, *p* = .03, *r* = .44, indicating the presence of a conflict adaptation effect for the error rates.

#### ERPs

A paired samples t-test performed over a 50 ms window between 150 and 200 ms post-stimulus onset for the collapsed posterior ROIs revealed a significant difference between post-congruent (M = -3.09, SD = 2.97) and post-incongruent trials (M = -2.66, SD = 2.89), *t*(21) = -3.163, *p* < .01, indicating a decrease in negative posterior activity after trials with conflict. Based on timing and topography, we interpret this negative-going component as a visual N1 component (Fig 3D). The inverse Bayes factor (BF_01_ = .05) for the effect of previous congruency in this experiment had a value below 1, providing 'strong' evidence in favor of the *alternative* hypothesis (i.e. in favor of the modulation).

### Time-frequency decompositions: Theta power

Because we found evidence for an attentional involvement in conflict adaptation in the Flanker task with predictable design, we did a time-frequency decomposition on this data to study oscillatory activity preceding and following this attentional modulation, and also provide this data for the random Flanker task for reference. We focused on these two experiments for the sake of simplicity, and because corresponding exploratory analyses for both Stroop experiments did not reveal a consistent result pattern.

**Fig 4:**
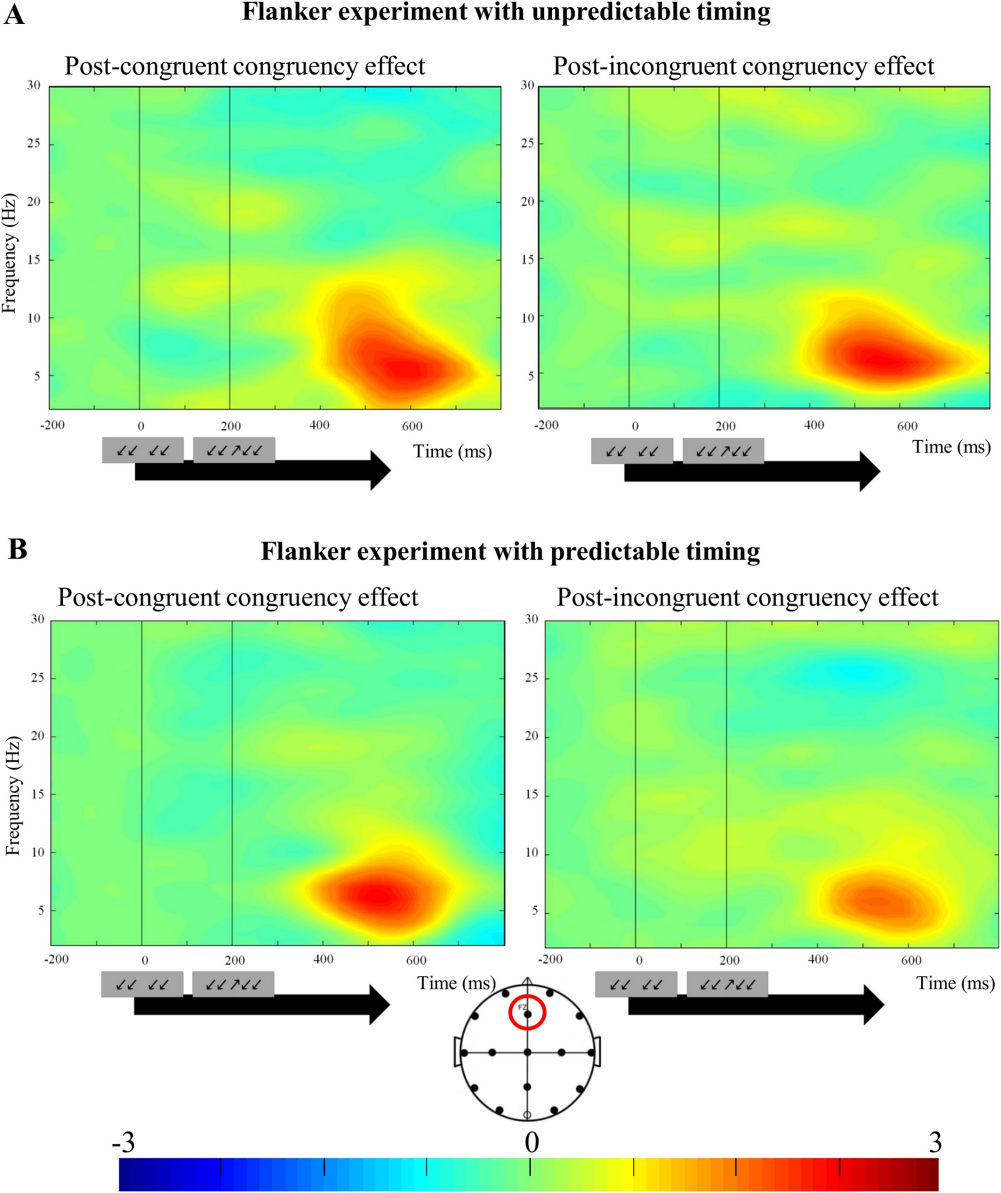
Theta power conflict adaptation effect in experiments 3&4—Flanker experiments with unpredictable and predictable timing. (A)(B) The theta power congruency effect (current incongruent trial minus current congruent trial) in irrelevant-first trials as a function of the congruency level of the previous trial in the Flanker experiments with unpredictable (A) and predictable (B) timing (4–8 Hz as the theta range, measured at Fz). We found evidence for a theta power conflict adaptation effect in the predictable, but not in the unpredictable Flanker experiment.

Many studies have found that increased oscillatory activity in the theta range (4–8 Hz), measured over fronto-midline scalp electrodes, reflects increased executive functioning during inhibitory processes [36–39]. With respect to conflict adaptation, some researchers found smaller mid-frontal theta power congruency effects after incongruent trials compared to congruent trials [40], whereas others did not [41]. Moreover, there is some evidence that higher parietal theta power during the inter-trial interval after incongruent trials can be interpreted as proactive adjustments of attentional control [40]. First, we analyzed theta power in the response-stimulus interval after trial N-1, i.e. before conflict adaptation occurs. A two-tailed paired samples t-test performed on mid-frontal theta power measurements over a 500 ms window between 500 and 1000 ms after the response on trial N-1 (measured at Fz) revealed a highly significant difference between post-congruent (M = -0.73, SD = 1.73) and post-incongruent response-stimulus intervals or RSIs (M = .34, SD = 1.32), *t*(21) = -2.90, *p* = .008. On average, theta power increased significantly more after incongruent trials than after congruent trials. Next, we looked at conflict-related theta power in the current trial (Fig 4). A repeated-measures ANOVA with factors previous congruency and current congruency performed over a 100-ms window between 500 and 600 ms after the onset of the irrelevant stimulus information (again measured at FCz) showed a marginally significant main effect of previous congruency, *F*(1,21) = 3.90, *p* = .06, *r* = .40, and a highly significant main effect of current congruency, *F*(1,21) = 21.48, *p* < .001, *r* = .72. Interestingly, there was a significant interaction between previous and current congruency, *F*(1,21) = 5.76, *p* = .03, *r* = .47, showing the presence of a conflict adaptation effect for mid-frontal theta power (CC trials = .43 dB; CI trials = 2.21 dB; IC trials = .39 dB; II trials = 1.72 dB).

Although we could not identify a neural marker of an attentional contribution to conflict adaptation in the Flanker experiment with an unpredictable design we also probed those data for the above theta effects. For comparability, we only analyzed trials in which the irrelevant information was presented first (just like in the experiment with a predictable design). A two-tailed paired samples t-test performed on mid-frontal theta power in the response-stimulus interval after trial N-1 between 500 and 1000 ms after the response (measured at Fz) showed no significant differences between post-congruent (M = -.89, SD = .93) and post-incongruent RSIs (M = -.97, SD = 1.12), *t*(21) = .22, *p* = .83. With respect to the conflict-related theta power in the current trial, a repeated-measures ANOVA with factors previous congruency and current congruency performed over a 100-ms window between 500 and 600 ms after the onset of the irrelevant stimulus information (measured at FCz) showed no significant main effect of previous congruency, *F*(1,21) = .01, *p* = .94, *r* = 0, but a highly significant main effect of current congruency, *F*(1,21) = 24.93, *p* < .001, *r* = .74 (more theta power for current incongruent trials). There was no significant interaction between previous and current congruency, *F*(1,21) = .03, *p* = .87, *r* = .001, showing the lack of a conflict adaptation effect for mid-frontal theta power (CC trials = .02 dB; CI trials = 1.85 dB; IC trials = .05 dB; II trials = 1.85 dB).

## General discussion

The main objective of this study was to find early attentional EEG markers of conflict adaptation by looking at the effect of previous conflict on early attentional stimulus processing in an optimized paradigm to disentangle specific effects on task-relevant and -irrelevant information processing. Such modulations are generally expected based on theoretical accounts of cognitive control [for a review, see 1, 2], but evidence from techniques with high temporal resolution is scarce. Based on this background, we expected that after an incongruent trial less attention would be deployed to the irrelevant stimulus dimension when presented shortly before the relevant dimension, whereas more attention would be deployed in case the relevant dimension was presented before the irrelevant dimension, since previous fMRI studies found evidence for both mechanisms [13, 16, 17]. Yet, we found such modulations only in one out of four experiments, namely in a Flanker task with a predictable irrelevant-first temporal arrangement. More specifically, we observed a posterior modulation reflecting a decreased negativity starting around 150 ms that could be viewed as a decreased visual N1 component based on timing and topography or a (conceptually related) selection negativity [21]. It is important to note that this modulation took place before the processing of the relevant dimension (i.e. target arrow) began, making it a specific measurement of post-conflict attentional inhibition of irrelevant information. Moreover, this attentional modulation was accompanied by modulations in inter-trial mid-frontal theta power and a theta-power conflict adaptation effect. Therefore, we interpret these results as evidence for an adaptive mechanism based on relative attentional inhibition.

The fact that we found conflict-adaptation-related attentional modulations in only one of the four experiments was surprising given not just the role they play in theoretical accounts of conflict adaptation but also numerous fMRI findings that are consistent with such an account [13–17]. Given fMRI’s limited temporal resolution, however, data with a higher temporal resolution would be desirable, but the empirical wealth of fMRI work is not equaled in human EEG research, with only two studies explicitly looking at attention in conflict adaptation. Scerif et al. [18] found evidence for a selective enhancement of the visual P1 component for incongruent trials when preceded by incongruent trials in a standard flanker task. More related to this study, when no-target flanker trials were preceded by incongruent trials, they observed a smaller P1 component, which they explained as more focused spatial attention. Similarly, Suzuki and Shinoda [19] showed decreased N1 amplitudes for regular Flanker trials after incongruent trials. The crucial difference between these previous studies and our study is our attempt to systematically disentangle modulations of task-relevant and task-irrelevant information by presenting irrelevant information randomly shortly before, at the same time, or briefly after the presentation of the relevant dimension. The fact that we only found the expected modulations in a Flanker task with a predictable irrelevant-dimension-first arrangement might, in part, relate to the fact that it has been suggested that conflict adaptation has a prominent proactive component (i.e., reflecting the expected nature of the n trial, rather than just reactively to that of the n-1 trial). This would depend on the ability or tendency of predicting the features of the subsequent trial [2, 42], which is less possible in experiments with unpredictable timing in the present study. As for the experiments with predictable timing, we limited ourselves to the irrelevant-first condition for the reason that this set-up yields strong conflict adaptation, which relevant- first does not [20, 43], and still splits out one aspect of stimulus processing, which simultaneous presentations do not. If attentional adjustments occur reactively, we hypothesized that such mechanisms would also be visible in the task variants with unpredictable timing, which, however, we did not observe. Yet, this choice obviously limits our ability to diagnose possible effects of attentional enhancement of relevant information to the random-SOA context, which by itself is rather atypical. Still, our results favor the notion that the attentional modulation we found in a predictable context probably does not occur in a purely reactive way and cannot easily be generalized to other contexts.

Interestingly, we did find a significant behavioral conflict adaptation effect in all four experiments, but only for trials in which the irrelevant flanker distractors or word names were presented 200 ms before the relevant target. The fact that there was a significant conflict adaptation effect in both experiments with predictable distracter-first trials is very much in line with the studies by Weisman and colleagues [44, 45] who also found that conflict adaptation is larger when irrelevant distracter information is always presented before the target information. With respect to the lack of conflict adaptation in relevant-first and simultaneous trials, it is possible that participants simply ended up experiencing very little cognitive conflict on trial n, leading to an absence of conflict adaptation effects. This would be in line with the findings of Appelbaum and colleagues [43], showing much larger congruency effects for trials in which irrelevant information was presented first (but see [46] for alternative findings). Therefore, the absence of conflict adaptation on the behavioral level for trials with simultaneous and relevant-first presentation in both Stroop and Flanker experiments seems to reflect a context effect, which abolishes conflict adaptation in a standard condition with simultaneous presentation of relevant and irrelevant stimulus information, likely through a shift from reactive to proactive control processes. However, it is important to note that the behavioral measures of conflict adaptation were of limited interest to this study, since they are also determined by the current congruency status of the trial and do not reflect the processing of irrelevant or relevant information in an isolated way.

A possible explanation why the attentional modulation only occurred in the Flanker task might be related to differences in attentional mechanisms. Specifically, the Flanker task emphasizes spatial attention, whereas the Stroop task would seem to be more related to feature attention [17]. It might be the case that the modulation we found is only related to spatial filtering of the visual field (after conflict, less attention is allocated to the visual field on the left and right of the target location), which can explain its absence in the Stroop task. If we relate these considerations back to the much more developed area of fMRI evidence for attentional modulations, it is worth noting that a large number of these studies used variants of the Stroop task that present relevant information in the form of categories for which specialized processing modules exist (often faces). It seems possible that such a set-up is more amenable to attentional modulations, although with fMRI it is impossible to determine their precise timing. Given that the Stroop task is likely more related to feature attention, it is possible that we simply failed to detect this kind of attention because it does not show up easily in the ERP.

When extending our analysis to theta power modulations, we found evidence for a theta power conflict adaptation effect in the predictable, but not in the unpredictable Flanker experiment. Notably, previous research has reported mixed findings on this issue. Pastotter et al. [40] conducted a response-priming task and did observe a current trial conflict adaptation effect in mid-frontal theta power. Moreover, they found that this theta power adaptation effect correlated with the behavioral conflict adaptation effect. In contrast, Cohen and Cavanagh [41] only found a theta power conflict adaptation effect on the single-trial level but failed to find it on the trial-averaged level. It was suggested that this effect might be harder to find in tasks with both stimulus and response conflict like the Stroop and Flanker task than in response-priming task with only response conflict. Nevertheless, we did find the effect and the fact that the theta power evidence for conflict adaptation is only present in the experiment with the attentional modulation supports the notion that it can indeed be a neural marker of the post-conflict adaptation mechanism (i.e. inhibition of irrelevant information) in a predictable context. This raises the possibility that features related to the predictability of stimulus features play a role in whether or not such a neural conflict adaptation pattern is observed or not. Furthermore, we observed increased post-conflict inter-trial theta power, time-locked to the response of trial n-1 in the predictable, but not in the unpredictable Flanker experiment. Pastotter et al. [40] also observed higher parietal theta power during the inter-trial interval after incongruent trials, which they interpreted as proactive adjustments of attentional control. Our finding of such a modulation in the task context that also showed an attentional modulation of the attentional processing of the subsequent trial is therefore highly consistent with this notion.

Finally, not controlling for feature integration [5] and contingency learning [47] processes in the different experiments is likely a limitation of the present study, as far as disambiguation along such lines goes. The reason why we intentionally did not control for these confounds has to do with the fact that recent accounts of conflict adaptation argue against a strict distinction between feature integration, contingency learning, and other types of cue learning, as learning itself is considered the mechanism behind cognitive control [48, 49]. Within this new perspective, it would even be artificial to exclude all learning possibilities and design an experiment where trial n shares no features of trials n-1 [50]. In other words, when taking away more learning possibilities, we would have reduced conflict adaptation, making it harder to find ERP markers of underlying neural processes. So although it is true the present findings cannot unambiguously discriminate between cognitive control and learning processes, we see those as inherently related, and we do not think this invalidates the conclusions we make on the underlying mechanisms of the conflict adaptation effect.

## Conclusion

In sum, although we expected to find both a decrease in attentional allocation for irrelevant distractor information and an increase for relevant target information during conflict adaptation, we could only identify the former, and only in a very specific task context of a Flanker task with a temporal arrangement in which the irrelevant stimulus dimension was always presented first. Given this specificity, our experiments emphasize the role of strategic top-down processes.
